# Deep learning-based image analysis for automated measurement of eyelid morphology before and after blepharoptosis surgery

**DOI:** 10.1080/07853890.2021.2009127

**Published:** 2021-11-30

**Authors:** Lixia Lou, Jing Cao, Yaqi Wang, Zhiyuan Gao, Kai Jin, Zhaoyang Xu, Qianni Zhang, Xingru Huang, Juan Ye

**Affiliations:** a Department of Ophthalmology, School of Medicine, The Second Affiliated Hospital of Zhejiang University, Hangzhou, China; b College of Media Engineering, Communication University of Zhejiang, Hangzhou, China; c Wellcome-MRC Cambridge Stem Cell Institute, University of Cambridge, Cambridge, UK; d School of Electronic Engineering and Computer Science, Queen Mary University of London, London, UK

**Keywords:** Blepharoptosis, eyelid morphology, automated measurement, deep learning, surgical outcome

## Abstract

**Background and aim:**

Eyelid position and contour abnormality could lead to various diseases, such as blepharoptosis, which is a common eyelid disease. Accurate assessment of eyelid morphology is important in the management of blepharoptosis. We aimed to proposed a novel deep learning-based image analysis to automatically measure eyelid morphological properties before and after blepharoptosis surgery.

**Methods:**

This study included 135 ptotic eyes of 103 patients who underwent blepharoptosis surgery. Facial photographs were taken preoperatively and postoperatively. Margin reflex distance (MRD) 1 and 2 of the operated eyes were manually measured by a senior surgeon. Multiple eyelid morphological parameters, such as MRD1, MRD2, upper eyelid length and corneal area, were automatically measured by our deep learning-based image analysis. Agreement between manual and automated measurements, as well as two repeated automated measurements of MRDs were analysed. Preoperative and postoperative eyelid morphological parameters were compared. Postoperative eyelid contour symmetry was evaluated using multiple mid-pupil lid distances (MPLDs).

**Results:**

The intraclass correlation coefficients (ICCs) between manual and automated measurements of MRDs ranged from 0.934 to 0.971 (*p* < .001), and the bias ranged from 0.09 mm to 0.15 mm. The ICCs between two repeated automated measurements were up to 0.999 (*p* < .001), and the bias was no more than 0.002 mm. After surgery, MRD1 increased significantly from 0.31 ± 1.17 mm to 2.89 ± 1.06 mm, upper eyelid length from 19.94 ± 3.61 mm to 21.40 ± 2.40 mm, and corneal area from 52.72 ± 15.97 mm^2^ to 76.31 ± 11.31mm^2^ (all *p* < .001). Postoperative binocular MPLDs at different angles (from 0° to 180°) showed no significant differences in the patients.

**Conclusion:**

This technique had high accuracy and repeatability for automatically measuring eyelid morphology, which allows objective assessment of blepharoptosis surgical outcomes. Using only patients’ photographs, this technique has great potential in diagnosis and management of other eyelid-related diseases.

## Introduction

The eyelid acts as a protective layer of the eyeball and is vital to maintain a healthy ocular surface through regular blinking [1]. Eyelid position and contour abnormality could lead to various diseases, such as blepharoptosis, which is common among patients presenting for oculoplastic surgery [2]. Accurate assessment of eyelid morphology is necessary for the diagnosis of blepharoptosis, surgical planning, and evaluation of surgical outcomes. Margin reflex distance 1 and 2 (MRD1 and MRD2) are the most common indicators of eyelid position [3]. Manual measurement of MRD performed by a skilled clinician using a penlight and a ruler could yield reliable results [4]. However, the accuracy of manual measurements relies on the experience of clinicians, and in some cases, measurement of eyelid heights is challenging [5]. In our previous study, we proposed a novel approach for automated measurement of MRDs in blepharoptosis patients using digital image analysis [6]. This technique allows objective assessment of the degree of blepharoptosis with high accuracy.

As is known to all, MRDs alone are not enough to define morphological properties of eyelids [7]. Recent studies have attempted to evaluate eyelid contours, two-dimensional measurements (such as exposed corneal area), or other specific parameters (such as eyelid length) based on facial image [8–11]. However, both inter- and intra-observer variabilities inevitably exist in image measurement in the context of human–computer interaction. An optimized automated system with comprehensive investigation of eyelid morphological properties would provide a more accurate tool for oculoplastic research and clinics. Deep learning with convolutional neural networks (CNN) has achieved state-of-the-art performance for automatic ophthalmological image segmentation [12]. Traditionally, the assessment of surgical effectiveness in eyelid morphology was purely subjective, typically including descriptors such as “excellent”, “good”, “fair” or “poor”. So far none of the studies presented clear comparisons of preoperative eyelid morphology and postoperative results following blepharoptosis surgery. Therefore, the aims of this study were to propose a novel deep learning-based image analysis to automatically measure eyelid morphological properties before and after blepharoptosis surgery, as an objective evaluation of surgical effectiveness.

## Materials and methods

### Ethical approval

This study was approved by the Institutional Review Board of the Second Affiliated Hospital of Zhejiang University, School of Medicine, Hangzhou, China (approval number: 2020-583). Informed consent was obtained from patients aged ≥ 18 years and guardians of those aged < 18 years, in accordance with the Declaration of Helsinki.

### Study participants

Patients who underwent blepharoptosis surgery in our oculoplastic clinic between January 2016 and September 2019 were invited to participate in this study. The surgeries were performed by a senior surgeon (J.Y.) who had more than 15 years of experience in oculoplastic surgery. Exclusion criteria were: variable ptosis (e.g. myasthenia gravis), coexisting eyelid diseases (e.g. entropion, ectropion, enophthalmos, or exophthalmos), strabismus, and abnormalities of pupil.

In total, 103 patients (135 ptotic eyes) including 71 patients with unilateral blepharoptosis and 32 patients with bilateral blepharoptosis were included in this study. seventy-three patients were male and 30 patients were female. The mean age was 6.3 years old, ranging from 1 to 50 years old. 117 eyes underwent frontalis suspension, 17 eyes underwent levator resection, and one eye underwent levator aponeurosis repair. The mean follow-up duration was 21 weeks, ranging from 2 to 109 weeks.

### Manual measurements and image collection

Preoperative and postoperative MRD1 and MRD2 of the operated eyes were manually measured by a senior surgeon (J.Y.). When the patients were gazing in the primary position, a penlight was used to produce the corneal light reflex and a ruler was used to judge the vertical distance from the corneal light reflection to the upper eyelid margin (MRD1) and to the lower eyelid margin (MRD2). If the upper eyelid covered the corneal light reflection, then the examiner raised the eyelid until the reflection was seen. The number of millimetres the eyelid must be raised was recorded as the MRD1 in negative numbers.

Facial photographs were taken preoperatively and at the last follow up. A circular marker with a diameter of 10 mm was placed on the forehead of the patients. The patients were asked to gaze in the primary position and a digital camera Canon 500 D (Canon Corporation, Tokyo, Japan) was positioned at eye level at a distance of 1 m.

### Image analysis

The automated image analysis for comparisons of preoperative and postoperative eyelid morphology included the following four steps.

Step 1: Regions of eyes were localized by an open-source project named Face Alignment [13]. Facial image of 2069 volunteers (4138 eyes) from the Second Affiliated Hospital of Zhejiang University were used to train the eye segmentation network. The eyelid and the corneal limbus were outlined by two ophthalmologists. The pre-processing methods, such as random noise, colour perturbation, random scaling and elastic transformation, were used. Then, the regions of interest were sent into the Attention Recurrent Residual Convolutional Neural Network based on U-Net (Attention R2U-Net) [14]. Parameter settings of the network model: epoch = 200; batch size = 4; input image size = 256 × 256 pixels; logistic loss function: L1 loss; optimiser: Adam (lr = 0.00001).

Step 2: Preoperative and postoperative facial images of 103 patients (135 operated eyes) who underwent blepharoptosis surgery were used as the test set. The Attention R2U-Net based model predicted eyelid and cornea segmentation mask. Three points were randomly chosen from the cornea margin to fit the cornea circle and the circle centre was set as the pupil centre. Since the cornea is not perfectly round, the process was repeated 2000 times and the final pupil centre was localized by the mean shift algorithm with Gaussian kernel [15].

**Figure 1.**
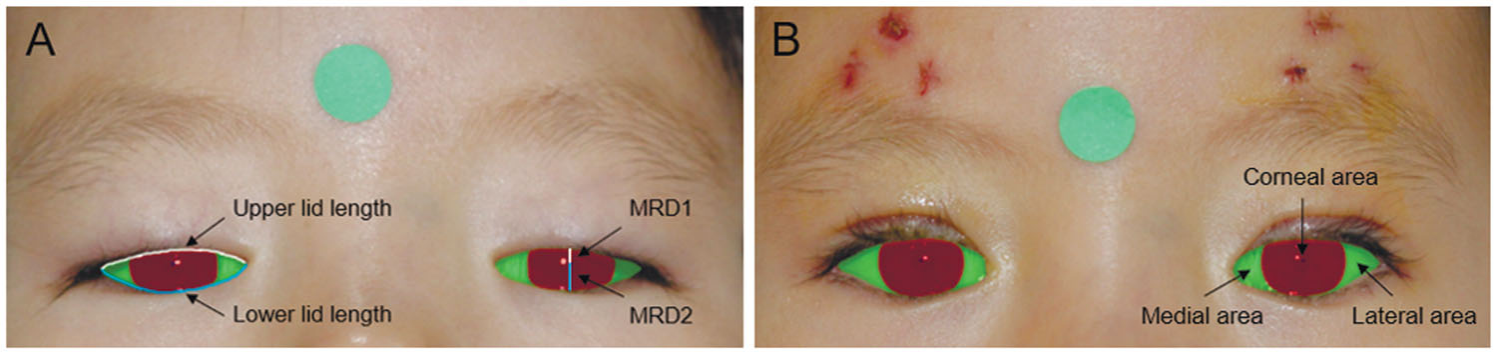
The automatically segmented eye images in a same patient (A) pre-operatively and (B) post-operatively. Corneal area was marked in red colour, and medial area and lateral area were marked in green colour. Pupil centre was marked with a blue dot. MRD1 and MRD2 were marked with a white and a blue straight line. Upper lid length and lower lid length were marked with a white and a blue curve.

**Figure 2.**
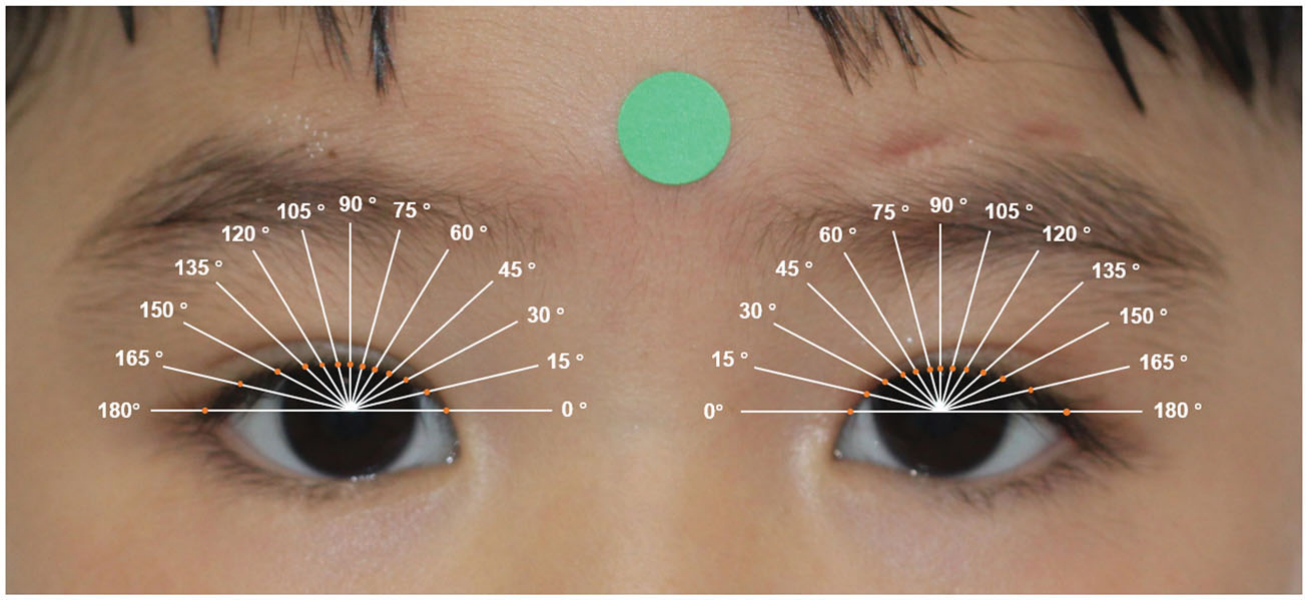
Post-operative multiple lid lines equally angularly (15°) spaced. Orange dots indicate the intersections of the radial lines on the lid margin. The radial MPLD lengths were calculated as the distance between the pupil centre and the orange dots.

Step 3: The pixel numbers of palpebral fissure length (including MRD1 and MRD2), lid length (including upper lid length and lower lid length), and palpebral fissure area (including medial area, corneal area, and lateral area) of the pre-operative and post-operative eyes were calculated (Figure 1). After location of the pupil centre, the conventional mid-pupil lid distance (MPLD) vertical line (90°) and 6 radial lines 15° apart from the midline in the nasal (0°, 15°, 30°, 45°, 60°, and 75°) and temporal (105°, 120°, 135°, 150°, 165°, and 180°) sectors of the lid fissure of the postoperative eyes were automatically drawn. The radial MPLD lengths were calculated as the distance between the pupil centre and the intersections of the radial lines on the lid margin (Figure 2) [8].

Step 4: Hough transform was used to detect the circular marker (10 mm in diameter) on the patient’s forehead [16]. Then, the pixel/millimetre ratios were calculated and the measurements of eyelid morphological parameters were converted into mm or mm^2^.

### Statistical analyses

Dice coefficient, as a statistical tool to measure the similarity of two samples, was calculated for the validation of eye segmentation algorithms [17]. Intraclass correlation coefficient (ICC), as a statistical tool to measure the reliability of an experimental method, was calculated for evaluating the agreement between manual and automated measurements, as well as the agreement between two repeated automated measurements of MRDs of the operated eyes preoperatively and postoperatively [18]. It was considered moderate agreement if 0.41 < ICC ≤ 0.60, substantial agreement if 0.60 < ICC ≤ 0.80, and excellent agreement if 0.80 < ICC ≤ 1.00. A Bland–Altman plot compares two assay methods. It plots the difference between the two measurements on the Y axis, and the average of the two measurements on the X axis [19]. Bland–Altman plots were used to visualize the differences between the two measurements (manual vs. automated; two repeated automated) of MRDs pre-operatively and post-operatively. Pre-operative and post-operative eyelid morphological parameters were compared with paired T-test. Post-operative eyelid contour symmetry was evaluated in patients with unilateral blepharoptosis and bilateral blepharoptosis respectively, using MPLDs. All statistical analyses were conducted using SPSS 23 (IBM Corporation, IL, USA). Statistical significance was set as 0.05.

## Results

### Agreement between two measurements of MRD1 and MRD2

**Figure 3.**
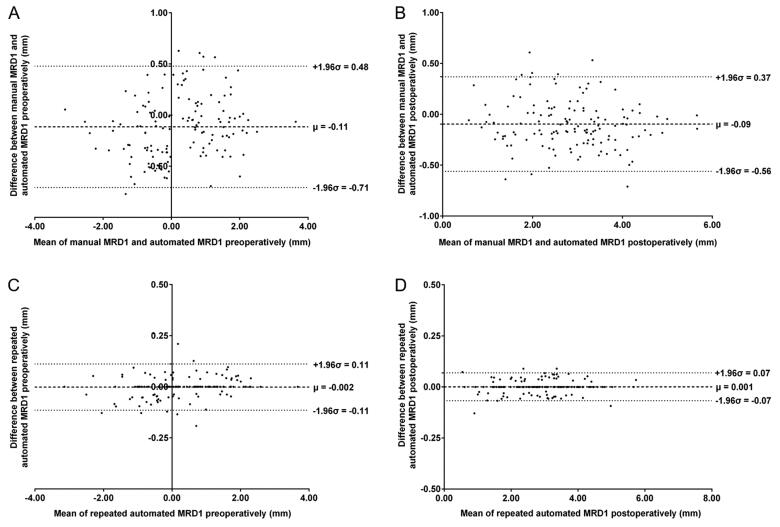
Bland–Altman plots analysing the agreement between two measurements of MRD1. (A) Agreement between manual MRD1 and automated MRD1 pre-operatively. (B) Agreement between manual MRD1 and automated MRD1 post-operatively. (C) Agreement between repeated automated MRD1 pre-operatively. (D) Agreement between repeated automated MRD1 post-operatively.

The dice coefficients for eye segmentation tasks in the test set were 0.962 for the eyelid and 0.964 for the cornea. Preoperative and postoperative manual and repeated automated measurements of MRD1 and MRD2 (mean ± standard deviation) in 135 ptotic eyes are shown in Table 1. Pre-operative manual measurements of MRD1 and MRD2 were 0.20 ± 1.22 mm and 5.61 ± 1.19 mm, respectively. Post-operative manual measurements of MRD1 and MRD2 were 2.80 ± 1.03 mm and 5.37 ± 0.84 mm, respectively. Repeated automated measurements were similar to the manual measurements. The ICCs between manual and automated measurements of MRDs ranged from 0.934 to 0.971 (all *p* < .001), indicating excellent agreement between the two methods (Table 2). The ICCs in MRD1 were better than that in MRD2. The ICCs between two repeated automated measurements of MRDs were up to 0.999 (all *p* < .001), which illustrated high repeatability of the automated method. The Bland–Altman plots also confirmed excellent agreement and acceptable limits of agreement between any two measurements for MRD1 (Figure 3) and MRD2 (Figure S1), with bias ranging from 0.09 mm to 0.15 mm between manual and automated measurements, and from 0.0002 mm to 0.002 mm between two repeated automated measurements. The scatter plots revealed that the difference between two measurements did not tend to get larger or smaller as the average increased, suggesting no relationship between difference and the level of measurements for MRDs.

**Table 1.** Pre-operative and post-operative manual and repeated automated measurements of MRD1 and MRD2 in 135 ptotic eyes.

	MRD1 (mm)	MRD2 (mm)
Preoperative		
Manual	0.20 ± 1.22	5.61 ± 1.19
Automated (1st)	0.31 ± 1.17	5.46 ± 1.16
Automated (2nd)	0.31 ± 1.16	5.46 ± 1.16
Postoperative		
Manual	2.80 ± 1.03	5.37 ± 0.84
Automated (1st)	2.89 ± 1.06	5.28 ± 0.81
Automated (2nd)	2.89 ± 1.06	5.28 ± 0.82

Mean ± standard deviation.

**Table 2.** Intraclass correlation coefficients between two measurements of MRD1 and MRD2 preoperatively and postoperatively.

	MRD1	MRD2
Preoperative		
Manual & Automated	0.964 (0.941–0.977)***	0.954 (0.916–0.973)***
Automated (1^st^) & Automated (2^nd^)	0.999 (0.998–0.999)***	0.999 (0.998–0.999)***
Postoperative		
Manual & Automated	0.971 (0.950–0.981)***	0.934 (0.901–0.955)***
Automated (1^st^) & Automated (2^nd^)	0.999 (0.999–1.000)***	0.999 (0.999–0.999)***

Intraclass correlation coefficient (95% confidence interval). ***p < 0.001.

### Pre- and post-operative eyelid morphological parameters comparisons

Preoperative and postoperative eyelid morphological parameters in 135 ptotic eyes are shown in Table 3. Paired T-tests revealed that all parameters, except MRD2, increased significantly after surgery. MRD2 showed no significant change postoperatively. Notably, MRD1 increased from 0.31 ± 1.17 mm to 2.89 ± 1.06 mm (*p* < .001), upper eyelid length from 19.94 ± 3.61 mm to 21.40 ± 2.40 mm (*p* < .001), and corneal area from 52.72 ± 15.97 mm^2^ to 76.31 ± 11.31mm^2^ (*p* < .001), which indicated great improvement of eyelid morphology after blepharoptosis surgery.

**Table 3.** Comparisons of preoperative and postoperative eyelid morphological parameters in 135 ptotic eyes.

Parameters	Preoperative	Postoperative
Palpebral fissure length (mm)	5.77 ± 1.39	8.17 ± 1.04***
MRD1 (mm)	0.31 ± 1.17	2.89 ± 1.06***
MRD2 (mm)	5.46 ± 1.16	5.28 ± 0.81
Lid length (mm)	40.87 ± 6.47	43.10 ± 4.71***
Upper lid length (mm)	19.94 ± 3.61	21.40 ± 2.40***
Lower lid length (mm)	20.93 ± 3.84	21.69 ± 2.35**
Palpebral fissure area (mm^2^)	87.41 ± 30.93	121.72 ± 24.37***
Medial area (mm^2^)	16.46 ± 11.48	19.81 ± 10.15***
Corneal area (mm^2^)	52.72 ± 15.97	76.31 ± 11.31***
Lateral area (mm^2^)	18.23 ± 10.80	25.60 ± 11.10***

Mean ± standard deviation. ***p < .001, **p < .01.

### Post-operative eyelid contour symmetry analyses

**Figure 4.**
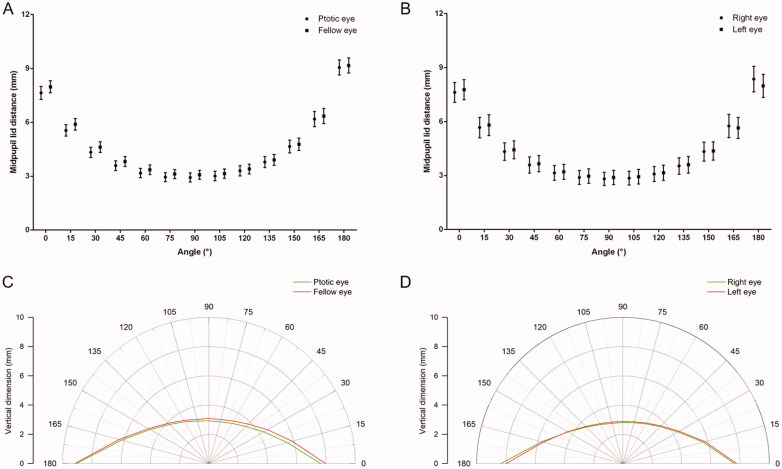
Post-operative mid-pupil lid distances (mean and 95% confidence interval) at different angles in (A) patients with unilateral blepharoptosis and (B) patients with bilateral blepharoptosis. Postoperative eyelid contour of (C) patients with unilateral blepharoptosis and (D) patients with bilateral blepharoptosis displayed on a polar plot.

Post-operative binocular eyelid morphological parameters in patients with unilateral blepharoptosis (*N* = 71) and patients with bilateral blepharoptosis (*N* = 32), respectively, are shown in Table S1. There were no significant differences in eyelid morphological parameters either between ptotic eyes and fellow eyes in patients with unilateral blepharoptosis, or between right eyes and left eyes in patients with bilateral blepharoptosis. Furthermore, postoperative binocular MPLDs at different angles (from 0° to 180°) were compared. Similarly, binocular MPLDs showed no significant differences in either patients with unilateral blepharoptosis or patients with bilateral blepharoptosis (Figure 4, Table S2), indicating ideal eyelid contour symmetry after surgery.

## Discussion

In this study, we introduced a deep learning-based image analysis system to automatically measure eyelid morphological properties in blepharoptosis patients. There was an excellent agreement between automated and manual measurements of MRD1 and MRD2. The findings also suggested high repeatability of automated measurements. Furthermore, pre-operative and post-operative eyelid morphology were compared and great improvement in MRD1, upper lid length and corneal area were found. In addition, postoperative eyelid contour using MPLDs showed perfect symmetry in all patients, indicating satisfactory outcomes after blepharoptosis surgery.

Traditionally, MRD is defined as the vertical distance from the corneal light reflection to the upper or lower eyelid margin, with the patient in primary gaze [3]. However, there is evidence of learning curve for MRD measurements. The difference of MRD measurements was up to 0.5 mm across clinicians with varying levels of experience, indicating slightly poor reproductivity [5]. In the present study, MRDs were measured by a senior surgeon with more than 15 years of experience in oculoplastic surgery following a standard measurement protocol, to minimize manual measurement error. The bias between manual and automated measurements of MRDs ranged from 0.09 mm to 0.15 mm, suggesting excellent agreement between the two methods. The critical steps to automatically measure eyelid features are accurate segmentation of eyelid margins and precise location of pupil centre. CNN-based deep learning has an advantage in eyelid segmentation over traditional edge detection algorithm, which is sensitive to insufficient contrast between eyelids and eyelashes [6,7]. Furthermore, two repeated automated measurements showed only the tiniest bias ranging from 0.0002 mm to 0.002 mm, which indicated high repeatability of the automated method.

Unlike previous studies which only focussed on linear measurements of eyelid position (such as MRDs) [4,6,7,20] and studies which assumed arbitrary corneal diameters as reference and reported virtual measurements [9,20–22], our study had attempted to measure multiple eyelid morphological properties using an actual marker sticked on the forehead. Chun et al. introduced a semi-automatic software to measure multiple features of normal eyelids [10]. Although they reported satisfactory intra-examiner reliability, the examiner needed to meticulously draw an eyelid border, which was a laborious task and time-consuming. In contrast, our algorithm automatically measuring eyelid features could save a lot of time and effort, especially when large volumes of images need to be analysed. Not surprisingly, MRD1 and corneal area increased after the drooping eyelid was elevated. Upper eyelid length also increased significantly, which may be largely attributed to the improvement of upper eyelid contour.

Some efforts had been made for measurement of eyelid contour [8,9,11,23–25]. Mocan et al. and Garcia et al. used polynomial functions to define eyelid contour, but the complex mathematical equations are unable to determine the real features and short of utility in clinical practices [9,24]. Additionally, their methods are limited because the eyelid in many pathologic conditions may not follow a uniform polynomial function. A more common method was calculating the distances from the centre of pupil to upper eyelid margin at 15° intervals from 0° to 180°, namely MPLDs, firstly proposed by Milbratz et al. [8]. MPLDs had been used to analyse eyelid contour in healthy volunteers [25], patients with Graves’ ophthalmopathy [26,27], and patients with blepharoptosis [23,28]. MPLDs allow quantitative analysis of eyelid contour and can easily be compared clinically across time points and groups of patients. The main disadvantage of MPLDs is the two-dimensional nature of the analysis. Three-dimensional anthropometry of eyelid contour may be more accurate [29,30]. It is also worth mentioning that MPLDs are unsuitable for use if a patient had negative MRD1. That is the reason why preoperative eyelid contour had not been plotted in our study.

Several limitations should be noted in this study. Firstly, our algorithm assumed that pupil and iris boundaries are perfect circles sharing the same centre, and hence, pupil centre was located only using circle fitting methods. Secondly, this system was only developed for 2 D photographs and had not been tested in 3 D photographs. Despite a typical flat forehead in Asian population, the eyelid and the circular marker on the forehead were not always at the same coronal position. In addition, the measurements of medial and lateral area of palpebral fissure would be smaller than the true value since 2 D photographs were used. Thirdly, this was a single-centre study only involving evaluation of surgical outcomes in blepharoptosis patients. Anthropometry in other eyelid-related disorders, such as Graves’ ophthalmopathy, is worthy for future exploration.

In conclusion, we proposed a novel image analysis technique to automatically measure eyelid morphological properties in blepharoptosis patients and to objectively evaluate surgical outcomes. Using only patients’ photographs, this technique could assist ophthalmologists with diagnosis, surgical planning and post-operative follow-up. The ease of accessing and transferring digital image also offers the possibility of telemedicine, benefiting people living in remote areas. Moreover, this new system would have wide potential application in other eyelid-related diseases, not limited to blepharoptosis.

## Consent for publication

This article includes photographs of individual participants and the authors have fully anonymized them. The authors confirm that consent to publish these details has been obtained from legal guardians of these individuals.

## Supplementary Material

Supplemental Material

## Data Availability

The original image data cannot be made publicly available because they contain identifying patient information. Data are available upon request from the Second Affiliated Hospital of Zhejiang University, School of Medicine.

## References

[1] Chang EI, Esmaeli B, Butler CE. Eyelid reconstruction. Plast Reconstr Surg. 2017;140(5):724e–735e. 10.1097/PRS.0000000000003820 29068942

[2] Liu CY, Chhadva P, Setabutr P. Blepharoptosis repair. Curr Opin Otolaryngol Head Neck Surg. 2018;26(4):221–226. 29746305 10.1097/MOO.0000000000000463

[3] Putterman AM. Margin reflex distance (MRD) 1, 2, and 3. Ophthalmic Plast Reconstr Surg. 2012;28(4):308–311. 10.1097/IOP.0b013e3182523b7f 22785597

[4] Nemet AY. Accuracy of marginal reflex distance measurements in eyelid surgery. J Craniofac Surg. 2015;26(7):e569–e71. 26468822 10.1097/SCS.0000000000001304

[5] Boboridis K, Assi A, Indar A, et al. Repeatability and reproducibility of upper eyelid measurements. Br J Ophthalmol. 2001;85(1):99–101. 11133723 10.1136/bjo.85.1.99 PMC1723668

[6] Lou LX, Yang LZ, Ye X, et al. A novel approach for automated eyelid measurements in blepharoptosis using digital image analysis. Curr Eye Res. 2019;44(10):1075–1079. 31148484 10.1080/02713683.2019.1619779

[7] Bodnar ZM, Neimkin M, Holds JB. Automated ptosis measurements from facial photographs. JAMA Ophthalmol. 2016;134(2):146–150. 26605967 10.1001/jamaophthalmol.2015.4614

[8] Milbratz GH, Garcia DM, Guimaraes FC, et al. Multiple radial midpupil lid distances: a simple method for lid contour analysis. Ophthalmology. 2012;119(3):625–628. 22197435 10.1016/j.ophtha.2011.08.039

[9] Mocan MC, Ilhan H, Gurcay H, et al. The expression and comparison of healthy and ptotic upper eyelid contours using a polynomial mathematical function. Curr Eye Res. 2014;39(6):553–560. 24401152 10.3109/02713683.2013.860992

[10] Chun YS, Hong HP, Park IK, et al. Topographic analysis of eyelid position using digital image processing software. Acta Ophthalmol. 2017;95(7):e625–e32. 28391655 10.1111/aos.13437

[11] Danesh J, Ugradar S, Goldberg R, et al. A novel technique for the measurement of eyelid contour to compare outcomes following muller's muscle-conjunctival resection and external levator resection surgery. Eye (Lond)). 2018;32(9):1493–1497. 29799019 10.1038/s41433-018-0105-4 PMC6137047

[12] Schmidt-Erfurth U, Sadeghipour A, Gerendas BS, et al. Artificial intelligence in retina. Prog Retin Eye Res. 2018;67:1–29. 30076935 10.1016/j.preteyeres.2018.07.004

[13] Kazemi V, Sullivan J. One millisecond face alignment with an ensemble of regression trees. 2014 IEEE Conference on Computer Vision and Pattern Recognition. 2014. 1867-74.

[14] Alom MZ, Yakopcic C, Taha T, et al. Nuclei segmentation with recurrent residual convolutional neural networks based U-Net (R2U-Net). NAECON 2018 - IEEE National Aerospace and Electronics Conference. 2018: 228-33.

[15] Aliyari Ghassabeh Y. A sufficient condition for the convergence of the mean shift algorithm with gaussian kernel. J Multivariate Anal. 2015;135:1–10.

[16] Zhang XQ, Qin SU, Jiang LY. Fast algorithm for circle detection using randomized hough transform. Comput Eng & Appl. 2006;44(22):62–64.

[17] Dalirsefat SB, da Silva Meyer A, Mirhoseini SZ. Comparison of similarity coefficients used for cluster analysis with amplified fragment length polymorphism markers in the silkworm, Bombyx mori. J Insect Sci. 2009;9:1–8. 10.1673/031.009.7101 PMC3011968 20050782

[18] Chen CC, Barnhart HX. Assessing agreement with intraclass correlation coefficient and concordance correlation coefficient for data with repeated measures. Comput Stat Data Anal. 2013;60:132–145.

[19] Bunce C. Correlation, agreement, and Bland-Altman analysis: statistical analysis of method comparison studies. Am J Ophthalmol. 2009;148(1):4–6. 19540984 10.1016/j.ajo.2008.09.032

[20] Nishihira T, Ohjimi H, Eto A. A new digital image analysis system for measuring blepharoptosis patients' upper eyelid and eyebrow positions. Ann Plast Surg. 2014;72(2):209–213. 23403544 10.1097/SAP.0b013e31825b8fb7

[21] Kim SS. Effects in the upper face of far east Asians after Oriental blepharoplasty: a scientific perspective on why Oriental blepharoplasty is essential. Aesthetic Plast Surg. 2013;37(5):863–868. 23979491 10.1007/s00266-012-9994-y

[22] Bravo FG, Kufeke M, Pascual D. Incidence of lower eyelid asymmetry: an anthropometric analysis of 204 patients. Aesthet Surg J. 2013;33(6):783–788. 23825308 10.1177/1090820X13495406

[23] Choudhary MM, Chundury R, McNutt SA, et al. Eyelid contour following conjunctival müllerectomy with or without tarsectomy blepharoptosis repair. Ophthal Plast Reconstr Surg. 2016;32(5):361–365. 10.1097/IOP.0000000000000545 26359699

[24] Garcia DM, Cruz AAV, Espirito Santo RO, et al. Lower eyelid contour in graves orbitopathy. Curr Eye Res. 2019;44(11):1216–1219. 31188037 10.1080/02713683.2019.1627460

[25] Lee H, Lee JS, Chang M, et al. Analysis of lid contour change with aging in Asians by measuring midpupil lid distance. Plast Reconstr Surg. 2014;134(4):521e–529e. 10.1097/PRS.0000000000000579 25357046

[26] Ribeiro SF, Milbratz GH, Garcia DM, et al. Pre- and postoperative quantitative analysis of contour abnormalities in graves upper eyelid retraction. Ophthalmic Plast Reconstr Surg. 2012;28(6):429–433. 23138202 10.1097/IOP.0b013e3182696532

[27] Ribeiro SF, Milbratz GH, Garcia DM, Fernandes VL, et al. Lateral and medial upper eyelid contour abnormalities in graves orbitopathy: the influence of the degree of retraction. Ophthalmic Plast Reconstr Surg. 2013;29(1):40–43. 23247033 10.1097/IOP.0b013e3182747537

[28] Ahn S, Lee H, Lee J, et al. Analysis of surgical outcome after levator advancement by assessing changes in eyelid contour. J Craniofac Surg. 2016;27(5):1147–1150. 27258709 10.1097/SCS.0000000000002694

[29] Malbouisson JM, Baccega A, Cruz AA. The geometrical basis of the eyelid contour. Ophthalmic Plast Reconstr Surg. 2000;16(6):427–431. 11106186 10.1097/00002341-200011000-00005

[30] Guo Y, Liu J, Ruan Y, et al. A novel approach quantifying the periorbital morphology: a comparison of direct, 2-dimensional, and 3-dimensional technologies. J Plast Reconstr Aesthet Surg. 2021;74(8):1888–1899. 33358464 10.1016/j.bjps.2020.12.003

